# CONVERSION THERAPY FOR GASTRIC CANCER: EXPANDING THE TREATMENT POSSIBILITIES

**DOI:** 10.1590/0102-672020190001e1435

**Published:** 2019-04-29

**Authors:** Marcus Fernando Kodama Pertille RAMOS, Marina Alessandra PEREIRA, Amir Zeide CHARRUF, André Roncon DIAS, Tiago Biachi de CASTRIA, Leandro Cardoso BARCHI, Ulysses RIBEIRO-JÚNIOR, Bruno ZILBERSTEIN, Ivan CECCONELLO

**Affiliations:** 1 Cancer Institute, University of São Paulo Medical School, São Paulo, SP, Brazil; 2 Hospital das Clínicas, Department of Gastroenterology, University of São Paulo Medical School, São Paulo, SP, Brazil

**Keywords:** Stomach neoplasms, Neoadjuvant therapy, Gastrectomy, Neoplasias gástricas, Terapia neoadjuvante, Gastrectomia

## Abstract

**Background:**

Conversion therapy in gastric cancer (GC) is defined as the use of chemotherapy/radiotherapy followed by surgical resection with curative intent of a tumor that was prior considered unresectable or oncologically incurable.

**Aim:**

To evaluate the results of conversion therapy in the treatment of GC.

**Methods:**

Retrospective analysis of all GC surgeries between 2009 and 2018. Patients who received any therapy before surgery were further identified to define the conversion group.

**Results:**

Out of 1003 surgeries performed for GC, 113 cases underwent neoadjuvant treatment and 16 (1.6%) were considered as conversion therapy. The main indication for treatment was: T4b lesions (n=10), lymph node metastasis (n=4), peritoneal carcinomatosis and hepatic metastasis in one case each. The diagnosis was made by imaging in 14 cases (75%) and during surgical procedure in four (25%). The most commonly used chemotherapy regimens were XP and mFLOX. Major surgical complications occurred in four cases (25%) and one (6.3%) died. After an average follow-up of 20 months, 11 patients (68.7%) had recurrence and nine (56.3%) died. Prolonged recurrence-free survival over 40 months occurred in two cases.

**Conclusion:**

Conversion therapy may offer the possibility of prolonged survival for a group of GC patients initially considered beyond therapeutic possibility.

## RESUMO

**Racional:**

A terapia de conversão no câncer gástrico (CG) é definida como o uso de quimio/radioterapia seguida de ressecção cirúrgica com intenção curativa de um tumor que era considerado irressecável ou oncologicamente incurável.

**Objetivo:**

Avaliar os resultados da terapia de conversão no tratamento do CG.

**Métodos:**

Análise retrospectiva de todas as operações de CG entre 2009 e 2018. Os pacientes que receberam alguma terapia antes da operação foram também identificados para definir o grupo de conversão.

**Resultados:**

Entre 1003 operações realizadas para o CG, 113 foram submetidos ao tratamento neoadjuvante e 16 (1,6%) considerados como terapia de conversão. As principais indicações para o tratamento foram: lesões T4b (n=10), metástase linfonodal (n=4), carcinomatose peritoneal e metástase hepática em 1 caso cada. O diagnóstico foi feito por exame de imagem em 14 casos (75%) e durante o procedimento cirúrgico em 4 casos (25%). Os esquemas quimioterápicos mais utilizados foram XP e mFLOX. Complicações cirúrgicas maiores ocorreram em 4 casos (25%) e 1 (6,3%) foi a óbito. Após seguimento médio de 20 meses, 11 pacientes (68,7%) apresentaram recidiva e 9 (56,3%) morreram. Sobrevida livre de recidiva prolongada acima de 40 meses ocorreu em dois casos.

**Conclusão:**

A terapia de conversão pode oferecer possibilidade de sobrevida prolongada para um grupo de pacientes com CG considerados inicialmente fora das possibilidades terapêuticas.

## INTRODUCTION

Gastric cancer (GC) is the fifth most common cancer in the world. It is estimated that almost one million (952,000) new cases occurred worldwide in 2012^11^. Surgery remains as the main curative treatment option, and gastrectomy with D2 lymphadenectomy is considered the standard surgical treatment for locally advanced GC. Unfortunately, many patients at the time of diagnosis have already locally unresectable tumors or signs of systemic disease^22^. For those clinical stage IV patients, palliative chemotherapy represents the current standard of care^18^. 

Recently, conversion therapy has emerged as an alternative therapy for these stage IV patients^26^. It consists in the administration of chemotherapy followed by surgery in stage IV patients. It is also referred as combination of induction chemotherapy and “adjuvant” surgery. This option can be indicated to treat unresectable or marginally resectable lesions, patients with distant lymph node metastasis (LNM) and even those with metastatic disease or peritoneal dissemination. In the last years, the development and improvement of chemotherapy regimens and molecular targeting agents based on molecular markers have improved dramatically the response rates^2^ ^,^ ^6^. Thus, it has become increasingly common for surgeons to reassess patients initially labeled as non-candidates for curative resection that present a complete different disease after initial palliative chemotherapy. This new scenario has brought conversion therapy to the primetime discussion of GC treatment. However, the clinical value of such multimodal strategy for stage IV GC remains controversial with few reports from western countries and very conflicting definitions of its use that may impair a clear analysis of its results. 

Thus, the aim of this study was to analyze the results of patients who were submitted to conversion therapy in our institution.

## METHODS

The study was approved by the hospital ethics committee (NP993/16) and registered online (www.plataformabrasil.com; CAAE: 2915516.2.0000.0065).

We reviewed our prospective database, selecting all patients submitted to any surgical procedure due to gastric adenocarcinoma from 2008 to 2018. Posteriorly, patients who received chemotherapy or radiotherapy followed by gastric resection were selected. Conversion therapy was defined as patients who were considered unresectable or marginally resectable and/or with disseminated disease during initial staging and were referred to initial chemo and/or radiation therapy. Those who had partial or complete response at re-assessment were indicated for surgery and considered as the conversion therapy group. 

Patients were staged preoperatively through abdominal and pelvis computed tomography, endoscopy and laboratory tests. Extension of gastric resection (total x subtotal) was based on the location of the tumor to obtain free proximal margin^27^. TNM staging was performed according to the TNM 7^th^ edition^24^. Clinical characteristics evaluated included American Society of Anesthesiologists (ASA) classification^8^, Charlson Comorbidity Index (CCI)^4^ and laboratory tests. CCI was considered without inclusion of GC as comorbidity. Surgical complications were graded according to Clavien-Dindo’s classification^7^. Major complications were considered Clavien III-V. Hospital length of stay and the number of retrieved lymph nodes were evaluated. Surgical mortality was considered when it occurred in the first 30 days after surgery or during hospital stay after the procedure.

The postoperative follow-up was performed on a quarterly basis in the first year and every six months in the following years. Follow-up tests for relapse detection were performed based on the presence of symptoms. Absence in consultations for more than 12 months was considered as loss of follow-up. All cases were operated in a high-volume center by specialized surgeons. The surgical technique, extension of resection and dissected lymph node chains followed the recommendations of the Japanese Gastric Cancer Association guidelines^18^. 

### Statistical analysis

The Chi-square test was used for categorical variables and t-tests for continuous variables. Overall survival (OS) and disease-free survival (DFS) were estimated using the method of Kaplan-Meier, and differences in survival were examined using the Log Rank Test. Survival time, in months, was calculated from the date of surgery until the date of death/recurrence. The patients alive were censored at the date of last contact. All tests were two-sided and *p*<0.05 was considered statistically significant. Analysis was performed using SPSS software, version 18.0 (SPSS Inc, Chicago, IL).

## RESULTS

Out of 1,003 GC patients operated in the period, surgical resection with curative intent was performed in 629 cases and palliative procedures in 230. A total of 113 patients were resected with curative intent after chemotherapy and/or radiotherapy. From this, 16 were considered as conversion therapy (1.6%).

Table 1 presents the clinicopathological characteristics of patients from the conversion group. Most patients had low ASA classification score (I-II) and CCI (0-1). Tumors were mainly located at the distal part of the stomach (56.3%) and intestinal adenocarcinoma was the most common histological subtype (43.8%). Considering the conversion group decision for chemo/radiotherapy was mainly based on radiologic exams (75%) and 4 patients (25%) were deemed unresectable/incurable during surgery. The chemotherapy regimens varied, with a predominance of schemes based on the combination of platin and fluoropyrimidine.

**TABLE 1:** Clinicopathological characteristics of conversion therapy

Variables		n = 16	%
Gender			
	Female	8	50
	Male	8	50
Age (years)			
	Mean (Range)	62.5 (48-80)	
Charlson Comorbidity Index (CCI)			
	0 - 1	10	62.5
	>=1	6	37.5
ASA			
	I-II	12	75
	III	4	25
Location of tumor			
	Upper	1	6.3
	Middle	4	25
	Lower	9	56.3
	Total	2	12.5
Histological type			
	Intestinal adenocarcinoma	7	43.8
	Diffuse adenocarcinoma	7	37.5
	Mixed adenocarcinoma	1	6.3
	Squamous cell carcinoma	1	6.3
Degree of histological differentiation			
	Well/ Moderately differentiated	9	56.3
	Poorly differentiated	7	43.7
Diagnosis of nonresectability			
	Surgery	4	25
	MRI	3	18.7
	CT	9	56.3
Preoperative treatment			
	Capecitabine + Cisplatin (XP)	5	31.3
	modified FLOX (mFLOX)	5	31.3
	Capecitabine + Oxaliplatin (Xelox)	1	6.3
	FOLFIRINOX	1	6.3
	Carboplatin + Paclitaxel	1	6.3
	Cisplatin + Iritonecan	2	12.5
	Radiotherapy (RDT)	1	6.3

Table 2 presents the surgical results. Combined organ resection was performed in 9 cases (56.3%) and in 4 of them more than 1 adjacent organ was resected. Liver and pancreas were resected in 5 cases and spleen and colon in 4. R0 resection was achieved in 13 cases (81.3%). The ypT4 category occurred in 8 patients (50%). The mean number of retrieved lymph nodes was 35.5, and 4 cases (25%) had no LNM. Only 2 cases were pathological stage IV. Four patients (25%) had major surgical complications and 1 (6.3%) died.

**TABLE 2:** Surgical results of conversion therapy

Variables		n = 16	%
Type of resection			
	Subtotal	8	50
	Total	8	50
Lymphadenectomy			
	D1	3	18.7
	D2	13	81.3
Combined ressection			
	No	7	43.8
	Yes	9	56.3
Residual disease			
	R0	13	81.3
	R1/R2	3	18.7
ypT			
	pT0/pT1	2	12.5
	pT2	1	6.3
	pT3	5	31.3
	pT4a	5	31.3
	pT4b	3	18.7
ypN			
	pN0	4	25
	pN1	5	31.3
	pN2	1	6.3
	pN3	6	37.5
ypTNM			
	I	1	6.3
	II	4	25
	III	9	56.3
	IV	2	12.5
Surgical complication			
	None / Clavien I - II	11	68.7
	Clavien III - IV	4	25
	Clavien V	1	6.3
Recurrence			
	No	5	31.3
	Yes	11	68.7
Death			
	No	7	43.8
	Yes	9	56.3

The median follow-up was 8.9 months (mean=16.2, Standard-Deviation=22.3). Eleven patients (68.8%) had recurrence and 9 (56.3%) died. Two patients had long-term survival without recurrence: one had local invasion to the pancreas and liver, and the other had invasion of the pancreas, duodenum and a gastrocutaneous fistula due to abdominal wall invasion. Characteristics of the patients and survival results are demonstrated in Table 3.

**TABLE 3:** Outcomes of conversion therapy

Case	Incurable factor	QT regimen	Surgery	Recurrence	Status	DFS*	OS*
1	T4b	XP	TG + D2	peritoneum/liver	loss of follow-up	14	21.7
2	LNM	mFLOX	STG + D2	peritoneum	dead	4.2	4.4
3	T4b	Cis + Irino	TG + D2	-	alive	91.3	91.3
4	T4b, LNM	RDT	TG + D1	-	dead	0.7	0.7
5	T4b, LNM	mFLOX	TG + D2	peritoneum/ LN	dead	7.4	9.8
6	T4b	mFLOX	TG + D1	-	alive	22.3	22.3
7	LNM	mFLOX	STG + D2	bone	dead	3.6	8
8	T4b	XELOX	STG + D2	-	alive	3	3
9	T4b, Carcinomatosis	XP	STG + D1	bone	dead	11.1	11.3
10	T4b, gastrocutaneous fistula	Cis + Irino	STG + D2	-	alive	40.6	40.6
11	LNM	XP	STG + D2	liver	dead	3.5	3.8
12	T4b, LNM	mFLOX	STG + D2	peritoneum	dead	5.3	5.8
13	LNM	XP	STG + D2	LN	alive	18.3	18.3
14	Lives metastasis	XP	TG + D2	liver	dead	0	16.2
15	T4b	Taxol + Carbo	TG + D2	liver / LN	alive	2.7	5.2
16	T4b, LNM	Folfirinox	TG + D2	peritoneum	dead	0	6.5

*months; TG= total gastrectomy; STG= subtotal gastrectomy

**FIGURE 1:**
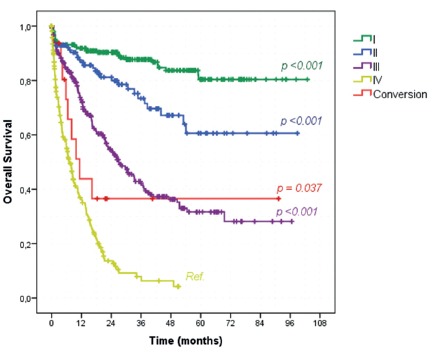
Kaplan-Meier overall survival curves according to the clinical stage compared to conversion therapy group

Survival analysis of all 1003 GC patients submitted to any surgical procedure demonstrated that, according to clinical stages, OS of the conversion group was higher than stage IV patients not submitted to conversion therapy (43.8% vs. 27%, *p=0.037*, Figure 1). The median OS for stage IV was 7 months compared to 11.3 months of the conversion group. Furthermore, there were no significant differences in the survival rates between stage III patients (52.3%, median OS=27 months) and the conversion group (*p=0.222*)

**FIGURE 2:**
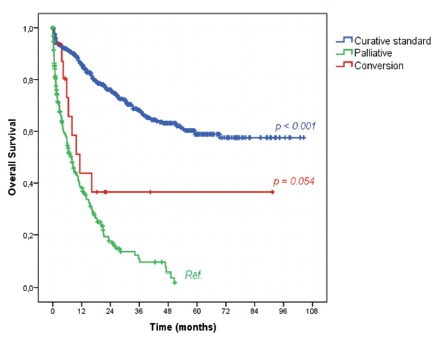
Kaplan-Meier overall survival curves according to the indication of surgical treatment

Regarding survival and the intention of the surgical procedures, patients who underwent conversion therapy had a trend to better OS than the ones submitted to palliative procedures (43.8% vs. 27.9%, *p=0.054*, Figure 2). The median OS was of 11.3 and 7.9 months for the conversion and palliative group, respectively. The standard curative treatment group had a significantly higher OS rate than palliative patients (served as reference group) with OS rate of 73.2% (*p<0.001*).

## DISCUSSION

Conversion therapy is an attempt to turn an incurable or unresectable/marginally resectable disease into a curable one. The concept and definition are often mixed and confused with other indications for GC management, especially with neoadjuvant chemotherapy. The last is indicated for resectable tumors aiming to downstage the lesion, reduce LNM and micrometastasis in order to improve survival. Palliative surgery is indicated based on the presence of symptoms, mainly bleeding and obstruction^14^. Cytoreductive surgery is the resection of an asymptomatic patient with disseminated disease^12^. Both cytoreductive and palliative are not intended to cure, but to improve the quality of life and/or prolong survival. The recent results of the REGATTA trial suggest that in metastatic patients cytoreductive surgery without prior chemotherapy did not offer benefits in survival compared with palliative chemotherapy^12^. Salvage surgery is the procedure indicated due to recurrence after a definitive chemotherapy and/or radiotherapy treatment. It is mainly related to esophageal tumors^21^.

It seems clear that the conversion therapy may have characteristics of all these definitions, but the main objective is to achieve a R0 resection and the cure of patients that were previously considerable incurable. Nevertheless, some controversies regarding its definition still persist. Some consider as conversion therapy any gastrectomy performed after prior palliative chemotherapy for unresectable GC^19^ ^,^ ^20^. Additionally, distant LNM most of the times are not technically unresectable, but they may also be included in the conversion group, similarly to the present study^9^ ^,^ ^20^. It is also extremely difficult to define what is marginally resectable or even unresectable tumor, and this varies a lot even among surgeons. This lack of a standardized definition makes it difficult to compare studies.

We were able to identify 16 patients who fitted the criteria for conversion therapy. Patients were younger with less comorbidities (most of them were ASA I-II with CCI of 0-1) than previous reports from our institution^23^. This reflects the ability of younger and healthier patients to endure the chemotherapy drawbacks optimizing its results to enable the surgical resection. Major complications occurred in 25% of the cases with 1 surgical death. Indeed, we expected a higher morbimortality rate due to the complexity of these procedures with 9 cases submitted to combined resection of other organs ^23^.

According to Yoshida *et al*.^26^ stage IV patients can be divided in four groups. The division is based on the presence of peritoneal disease, systemic metastasis, lymph node metastasis and resectability of the tumor. Type 1 tumors are defined as tumors oncologically stage IV, but with technically resectable metastasis without the need of any chemotherapy regimen to downstage the tumor. It is related mainly to single liver metastasis, positive peritoneal cytology and distant LNM. In this group, the administration of chemotherapy prior to surgery can be even considered as neoadjuvant. As this situation is not common, we consider it as conversion therapy in our analysis. Type 2 tumors have more than two liver metastasis, distant LNM or primary lesion larger than 5 cm located close to hepatic and/or portal vein.

 Patients with peritoneal dissemination (types 3 and 4) are considered to have the worst prognosis. In this case series, we only performed the procedure in one case with peritoneal metastasis with an unfavorable outcome. This poor result is also reported by other authors^9^ ^,^ ^25^. It must be highlighted that we did not added any kind of peritoneal chemotherapy to our procedure. Recently, the use of peritoneal chemotherapy and HIPEC has been attempt in this population^3^. Until now, there is no definite evidence of its effectiveness, but its use may increase the indication and the number of cases amenable to conversion therapy^1^ ^,^ ^17^.

We had two cases with favorable long-term results with OS over 40 months. They were both considered marginally resectable due to locally advanced tumors (Yoshida type 2). OS curves according to clinical stage demonstrated a slightly improvement of conversion therapy in relation to clinical stage IV tumors during the first two years. However, as we only have 16 cases of conversion therapy, those two cases long-term survivals play an important effect on the survival curve after two years. It even crosses the stage III curve. The same effect happens on the survival curve according to the intention of surgical treatment. Despite the statistical significance, it is possible to realize that the key point is to find out who is going to be the long-term survival. Otherwise, they will do slightly better than clinical stage IV and palliative procedure patients. 

Marginally resectable tumors are probably the most favorable indication for conversion therapy. However, it carries a high risk of classification bias. What is a marginally resectable tumor? A clear consensus and definition still lacks. Even for pancreatic cancer, that has been long using the term “borderline resectable”, there are some different definitions^16^. Thus, the inclusion of many “minor” marginally resectable tumors in the conversion therapy group may erroneously improve their outcomes. Additionally, the results of neoadjuvant therapy may also be falsely optimized by transferring these “borderline” patients to the conversion therapy group. This must be taken in account considering that most of the studies related to conversion therapy are retrospective. Therefore, the indication of preoperative chemotherapy, neoadjuvant or conversion, must be defined and reported before starting treatment.

Different regimens of chemotherapy were performed in our study. This reflects the different perspectives of the patients when they started palliative chemotherapy. The analysis of a period of nine years also plays a role in the variety of regimens adopted. Cisplatin and oxaliplatin, as well as 5-fluorouracil and capecitabine have been shown to be equally effective in advanced disease^5^. Cisplatin and irinotecan combination has demonstrated efficacy in a single arm phase II trial^15^ although this appears to be inferior than the platin and fluoropyrimidine combination in a randomized phase 2 study^10^. Given that there are several possible combinations, many factors should be take into account when choosing the chemotherapy backbone: comorbidities, performance status, infusion pump availability, ability to swallow tablets, availability to come to the center for treatment.

A major limitation of this study is the small number of patients included. Additionally, it is not possible to quantify the total of patients who underwent palliative treatment and could be considered as candidates for conversion. Therefore, the rate of patients who successfully complete the conversion therapy is unknown determining a relevant selection bias. Previous studies have reported rates between 26 and 32.4%^13^ ^,^ ^20^ ^,^ ^25^. Prospective trials with clear inclusion/exclusion criteria are needed to answer this question. A protocol of conversion therapy was recently in our institution designed to address this issue. Another limitation is that our palliative group, used in the survival analysis comparison, is formed only by patients submitted to surgical palliative procedures due to the presence of symptoms. Asymptomatic patients who received exclusive palliative chemotherapy were not included in the analysis.

In summary, our results suggest that conversion therapy should be considered with caution. The rational of conversion therapy and the reports of good clinical outcomes in these patients with limited perspectives encourages its promptly adoption. However, definitive unbiased data to corroborate its effectiveness and define the best candidates are still needed. Since the number of candidate patients for this therapy is too small to conduct a randomized clinical trial, the case series report, as our study, represents the current option to analyze and gather data.

## CONCLUSION

Conversion therapy may offer the possibility of surgical resection with long-term survival to a group of patients initially considered beyond therapeutic possibility. However, definitions regarding the best treatment regimen, diagnostic criteria of irresectability and which group of patients benefits from this modality are still necessary
